# Fruits and vegetables consumption and depressive symptoms: A population-based study in Peru

**DOI:** 10.1371/journal.pone.0186379

**Published:** 2017-10-12

**Authors:** Isabella Wolniczak, José A. Cáceres-DelAguila, Jorge L. Maguiña, Antonio Bernabe-Ortiz

**Affiliations:** 1 School of Medicine, Faculty of Health Sciences, Universidad Peruana de Ciencias Aplicadas–UPC, Lima, Perú; 2 CRONICAS Center of Excellence in Chronic Diseases, Universidad Peruana Cayetano Heredia, Lima, Perú; 3 Faculty of Epidemiology and Population Health, London School of Hygiene and Tropical Medicine, London, United Kingdom; Chiba Daigaku, JAPAN

## Abstract

**Objectives:**

Among different factors, diet patterns seem to be related to depression. The aim of this study was to evaluate the association between the consumption of fruits and/or vegetables and depressive symptoms.

**Methodology/Principal findings:**

A secondary data analysis was conducted using information from a population-based survey from 25 regions from Peru. The outcome was the presence of depressive symptoms according to the Patient Health Questionnaire (cutoff ≥15 to define major depressive syndrome); whereas the exposure was the self-reported consumption of fruits and/or vegetables (in tertiles and using WHO recommendation ≥5 servings/day). The association of interest was evaluated using Poisson regression models controlling for the complex-sample survey design and potential confounders. Data from 25,901 participants were analyzed, mean age 44.2 (SD: 17.7) and 13,944 (54.0%) women. Only 910 (3.8%; 95%CI: 3.5%–4.2%) individuals reported consuming ≥5 servings of fruits and/or vegetables/day; whereas 819 (2.8%; 95%CI: 2.5%–3.1%) had depressive symptoms. Those in the lowest tertile of fruits and/or vegetables consumption had greater prevalence of depressive symptoms (PR = 1.88; 95%CI: 1.39–2.55) than those in the highest tertile. This association was stronger with fruits (PR = 1.92; 95%CI: 1.46–2.53) than vegetables (PR = 1.42; 95%CI: 1.05–1.93) alone.

**Conclusions:**

An inverse relationship between consumption of fruits and/or vegetables and depressive symptoms is reported. Less than 5% of subjects reported consuming the amount of fruits and vegetables recommended by the WHO. There is a need to implement strategies to promote better diet patterns with potential impact on mental health.

## Introduction

Depression is a common mental disorder affecting approximately 350 million people worldwide and more than half of those do not receive treatment [1]. Moreover, depression is also recognized as the leading cause of disability and one of the greatest contributors to the global burden of disease [2].

There is an estimated 5% of people suffering from depression in low- and middle-income countries [1]. However, the burden of depression might change according to the population and the setting studied [3]. For example, a previous report found that the lifetime prevalence of depression in Peru was 6.4% according to the Worldwide Mental Health Study [4].

Depression is a multi-factorial disease attributable to social, environmental, genetic, physiological and biochemical factors [5]. Social variables associated with increased rates of depression include extreme poverty and overcrowding, amongst others [6, 7]. In addition, other common sociodemographic indicators associated with the presence of depression include variables such as: gender, age and marital status [8].

Among different factors, diet patterns seem to be related to depression. Previous studies have shown that individuals with a traditional diet based on the consumption of fish and fruits have lower risk of depressive symptoms than those with a diet high in fat and sugars [9, 10]. In a different study, a high vegetable intake was inversely associated with depression, but this relationship was not found with fruit or fish consumption [11]. On the contrary, the opposite relationship has been also established: people suffering from depression tend to have diets including lower rates of fruits and vegetables [12]. As a result, the potential influence of certain dietary patterns is still controversial.

Current changes in nutritional patterns seen in developing countries are partially influenced by the lifestyle from developed countries [13]; thus, recently Peru is considered as having the highest density of fast food restaurants in the world [14]. Therefore, the aim of this study was to assess the association between the consumption of fruits and vegetables and the presence of depressive symptoms, using data from a population-based study. Additionally, the prevalence of depression in our context was also estimated.

## Materials and methods

### Design and study area

A secondary data analysis was conducted using information from a population-based study in Peru. Data from the Health Questionnaire of the Demographic Health Survey (ENDES in Spanish) 2014 was utilized. This survey is performed annually collecting data from the 25 regions of Peru, including urban and rural settings [15]. The design of the survey included a multistage sampling strategy. In the rural areas, primary sampling units were villages of 500–2000 people and secondary sampling units were households within each of these villages. In the urban areas, sampling units consisted of blocks or groups of blocks with more than 2000 individuals, and a average of 100 houses and secondary sampling units were the same as in rural settings [16].

### Selection criteria

The ENDES survey usually includes residents from private households: women aged between 15 to 49 years and children under 5 years, as well as male individuals aged 15 years or above. Only the information collected from males or females that participated in the Health Questionnaire, aged ≥18 years, and habitual residents of the study setting selected was included for analyses.

### Definition of variables

The outcome of interest was depression, defined according to the Patient Health Questionnaire (PHQ-9) [17]. The questionnaire has been validated in Spanish in Chile [18] and adapted to the Peruvian population [19]. The tool includes nine questions to evaluate the presence of depressive symptoms in the last 14 days. Each question has four response options (never, some days, more than half of the day and nearly every day) and scored from 0 to 3; thus, the total score ranges from 0 to 27 points. For study purposes, a total score of 15 points or more was used to define depressive symptoms because it is indicative of a major depressive syndrome and requires treatment [17].

The exposure of interest was the self-reported consumption of fruits and vegetables, defined based upon the questions from STEP-wise approach to non-communicable disease risk factor surveillance (WHO STEPs) [20]. Fruits and vegetables consumption was assessed individually and together. In the latter case, two different definitions were utilized: the first one was based on the cut-point recommend by the WHO (i.e. five or more servings of fruit and vegetables per day–equivalent to 400 grams of fruits and vegetables per day) [21], and the second one was obtained by dividing the total number of fruits and vegetables calculated per day in tertiles (highest, middle and lowest).

Other variables considered into the analyses were gender; age group (18–34, 35–54, 55–74, and 75+ years); education level (< 7 years, 7–11 years, 12+ years); socioeconomic status, based on a wealth index created according to housing characteristics and assets, and then split into tertiles (low, middle or high); marital status (married, never married or previously married); region (coastal, highlands and jungle); place of residence (urban or rural); self-reported daily smoking (≥1 cigarette/day); binge drinking, defined as the consumption of alcohol for >12 days in the last 12 months; and previous treatment for depressive symptoms (yes vs. no), based on the question: “During the previous 12 months, have you received by any health professional, any treatment for depression, sadness, little interest, or irritability?”. Finally, hypertension status was also included, defined as systolic blood pressure ≥140 mmHg or diastolic blood pressure ≥90 mmHg or previous diagnosis by a physician.

### Procedures

This is a secondary analysis of a population-based survey freely available. The methods and procedures of data collection have been published elsewhere [15]. Briefly, data collection was performed using handheld computers (Personal Digital Assistants). Information was collected using a face-to-face technique. A field team was composed by a supervisor, two fieldworkers and an anthropometrist, which were trained before data collection activities.

### Sample size and power

With 25901 records available in the database of the ENDES, we have a power ≥80% to detect a prevalence ratio ≥1.7, assuming a prevalence of depressive symptoms of 6% [4], a proportion of individuals reporting a consumption of ≥5 servings of fruits and vegetables per day of 5% [22], and a design effect of 3 [23].

### Data analysis

Statistical analysis was performed with STATA 13 for Windows (StataCorp, College Station, TX, US). All analyses were adjusted for the complex-sample survey design considering the sample strata, primary sampling units and population weights [24].

The description of the study population was performed using proportions for categorical variables. Prevalence and 95% confidence intervals (95%CI) of exposure and outcome were reported. Chi square test was used for comparisons according to the variables of interest.

The association of interest was assessed using Poisson regression models [25], reporting prevalence ratios (PR) and 95%CI. Crude and adjusted models were built separately for fruits, vegetables and fruits and vegetables together. Gender was evaluated as potential effect modifier of the association of interest using the likelihood ratio test.

### Ethics

The research protocol was reviewed and approved by the Ethical Committee of the Universidad Peruana de Ciencias Aplicadas–UPC, Lima, Peru. This study included a secondary analysis of a de-identified database freely available and, as a result, the informed consent was waived by the Ethical Committee.

## Results

### Characteristics of the study population

Data from a total of 25,848 participants were included in the analyses. The mean age of participants was 44.2 (SD: 17.7) years, whereas 13,944 (54.0%) were women. The characteristics of the study population according to the consumption of fruits and vegetables (in tertiles) and taking into account the multistage study design are shown in Table 1 (un-weighted estimates are shown in S1 Table). Of interest, only daily smoking and binge drinking were not associated with the self-reported fruit and vegetables consumption.

**Table 1:** Characteristics of the study population according to fruits and vegetables consumption taking into account the complex sample design.

	Fruits and vegetables consumption (in tertiles)			
	Highest	Middle	Lowest	p-value^a^
	(n = 8,509)	(n = 8599)	(n = 8740)	
***Gender***				< 0.001
Male	3,517 (40.5%)	4,012 (46.9%)	4,375 (51.7%)	
Female	4,992 (59.5%)	4,587 (53.1%)	4365 (48.3%)	
***Age***				< 0.001
18–34 years	3,107 (39.7%)	3,018 (39.4%)	2,609 (34.5%)	
35–54 years	3,074 (36.9%)	3,083 (37.0%)	2,850 (36.3%)	
55–74 years	1,559 (18.5%)	1,642 (18.8%)	2,037 (21.8%)	
75+ years	401 (4.9%)	470 (4.8%)	874 (7.4%)	
Missing values	368	386	370	
***Education level***				< 0.001
< 7 years	2,383 (24.0%)	3,017 (28.5%)	4,483 (42.6%)	
7–11 years	3,323 (40.8%)	3,259 (40.6%)	2,776 (36.5%)	
12+ years	2,787 (35.2%)	2,299 (30.9%)	1,452 (20.9%)	
Missing values	16	24	29	
***Socioeconomic status***				< 0.001
Low	2,034 (14.4%)	2,516 (18.0%)	4,213 (34.1%)	
Middle	2,843 (27.6%)	3,112 (31.2%)	2,707 (30.3%)	
High	3,632 (58.0%)	2,971 (50.8%)	1,820 (35.6%)	
***Marital status***				0.002
Married	5,372 (61.7%)	5,472 (62.6%)	5,389 (63.0%)	
Never married	1,674 (25.9%)	1,641 (24.4%)	1,485 (21.8%)	
Previously married	1,463 (13.4%)	1,486 (13.0%)	1,866 (15.2%)	
***Region***				< 0.001
Coastal	3,855 (63.7%)	3,483 (59.0%)	2,712 (48.9%)	
Highlands	2,712 (22.7%)	3,498 (30.0%)	4,430 (39.0%)	
Jungle	1,942 (13.6%)	1,618 (11.0%)	1,598 (12.1%)	
***Place of residence***				< 0.001
Urban	6,010 (82.5%)	5,569 (77.8%)	4,343 (64.6%)	
Rural	2,499 (17.5%)	3,030 (22.2%)	4,397 (35.4%)	
***Daily smoking***				0.57
No	8,135 (97.9%)	8,430 (98.0%)	8,540 (97.6%)	
Yes	192 (2.1%)	165 (2.0%)	196 (2.4%)	
Missing values	2	4	4	
***Binge drinking***				0.87
No	7,477 (86.3%)	7,581 (85.9%)	7,776 (86.2%)	
Yes	1,022 (13.7%)	1,008 (14.1%)	945 (13.8%)	
Missing values	10	10	19	
***Previous depression***				0.02
No	8,120 (95.7%)	8,290 (95.9%)	8,500 (97.2%)	
Yes	386 (4.3%)	306 (4.1%)	236 (3.8%)	
Missing values	3	3	4	
***Hypertension status***				0.59
No	6,582 (77.4%)	6,682 (78.3%)	6,766 (78.2%)	
Yes	1,899 (22.6%)	1,887 (21.7%)	1,950 (21.8%)	
Missing values	28	30	24	

^a^ P-value was calculated using Chi squared test

On the other hand, when the demographic and behavioral variables were tabulated according to the presence of depressive symptoms, daily smoking and binge drinking were not associated with depression (Table 2). Un-weighted estimates are shown in S2 Table.

**Table 2:** Characteristics of the study population according to depressive symptoms taking into account the complex sample design.

	Depressive symptoms		
	No	Yes	p-value^a^
	(n = 25,029)	(n = 819)	
***Gender***			< 0.001
Male	11,683 (98.4%)	221 (1.6%)	
Female	13,346 (96.1%)	598 (3.9%)	
***Age***			< 0.001
18–34 years	8,584 (98.4%)	150 (1.6%)	
35–54 years	8,776 (97.3%)	231 (2.7%)	
55–74 years	4,992 (96.0%)	246 (4.0%)	
75+ years	1,594 (93.3%)	151 (6.7%)	
Missing values	1,083	41	
***Education level***			< 0.001
< 7 years	9,379 (95.4%)	504 (4.6%)	
7–11 years	9.142 (97.6%)	216 (2.4%)	
12+ years	6,447 (98.6%)	91 (1.4%)	
Missing values	61	8	
***Socioeconomic status***			< 0.001
Low	8,390 (96.4%)	373 (3.6%)	
Middle	8,401 (96.9%)	261 (3.1%)	
High	8,238 (97.7%)	185 (2.3%)	
***Marital status***			< 0.001
Married	15,818 (97.4%)	415 (2.6%)	
Never married	4,703 (98.3%)	97 (1.7%)	
Previously married	4,508 (94.4%)	307 (5.6%)	
***Region***			< 0.001
Coastal	9,795 (97.5%)	255 (2.5%)	
Highlands	10,200 (96.4%)	440 (3.6%)	
Jungle	5,034 (97.8%)	124 (2.2%)	
***Place of residence***			0.01
Urban	15,489 (97.4%)	433 (2.6%)	
Rural	9,540(96.6%)	386 (3.4%)	
***Daily smoking***			0.78
No	24,483 (97.2%)	802 (2.8%)	
Yes	537 (96.9%)	16 (3.1%)	
Missing values	9	1	
***Binge drinking***			0.03
No	22,072 (97.1%)	762 (2.9%)	
Yes	2,919 (98.1%)	56 (1.9%)	
Missing values	38	1	
***Previous depression***			<0.001
No	23,043 (97.6%)	674 (2.4%)	
Yes	787 (88.1%)	94 (11.9%)	
Missing values	10	0	
***Hypertension status***			<0.001
No	19,494 (97.5%)	536 (2.5%)	
Yes	5,458 (96.1%)	278 (3.9%)	
Missing values	77	5	

^a^ P-value was calculated using Chi squared test

### Consumption of fruits and vegetables and depressive symptoms

Only 910 (3.8%; 95%CI: 3.5%–4.2%) individuals reported consuming ≥5 servings of fruits and vegetables per day. On the other hand, 819 (2.8%; 95%CI: 2.5%–3.1%) individuals were categorized as having depressive symptoms. Interestingly, 928 (3.9%; 95%CI: 3.5%–4.2%) subjects of the total sample reported having received previous treatment for depressive symptoms.

There was a clear increasing trend in the prevalence of depressive symptoms according to the consumption of fruits (highest tertile: 1.8% vs. lowest tertile: 3.8%; p<0.001), the consumption of vegetables (highest tertile: 2.3% vs. lowest tertile: 3.8%; p = 0.003), and the combined consumption of fruits and vegetables (highest tertile: 1.9% vs. lowest tertile: 4.0%; p<0.001).

In multivariate model, there was evidence of strong association between the presence of depressive symptoms and the consumption of fruits, vegetables and fruits and vegetables together (Table 3 and S3 Table). Gender was not an effect modifier of the association of interest. In addition, when the cutoff of ≥5 servings of fruits and vegetables was used as defined by the WHO, there was association between the exposure and depressive symptoms (PR = 0.50; 95%CI: 0.28–0.87) in the crude model, but this was in the limit of significance after controlling for several potential confounders (PR = 0.58; 95%CI: 0.33–1.02).

**Table 3:** Association between fruits and vegetables consumption and depressive symptoms: Crude and adjusted models taking into account the complex sample design.

	Depressive symptoms		Crude model	Adjusted model^a^	Adjusted model^b^
*Consumption of*.* *.* *.	No (n = 25,029)	Yes (n = 819)	PR (95%CI)	PR (95%CI)	PR (95%CI)
**.* *.* *.*fruits***					
Highest	7,724 (98.2%)	187 (1.8%)	1 (Reference)	1 (Reference)	1 (Reference)
Middle	8,600 (97.2%)	258 (2.8%)	1.55 (1.16–2.07)	1.41 (1.05–1.90)	1.47 (1.09–1.98)
Lowest	8,705 (96.2%)	374 (3.8%)	2.07 (1.62–2.66)	1.81 (1.37–2.39)	1.92 (1.46–2.53)
**.* *.* *.*vegetables***					
Highest	6,842 (97.7%)	178 (2.3%)	1 (Reference)	1 (Reference)	1 (Reference)
Middle	9,729 (97.5%)	244 (2.5%)	1.07 (0.79–1.47)	1.01 (0.73–1.41)	1.01 (0.73–1.40)
Lowest	8,458 (96.3%)	397 (3.8%)	1.62 (1.22–2.15)	1.39 (1.02–1.89)	1.42 (1.05–1.93)
**.* *.* *.*fruits and vegetables***					
Highest	8,311 (98.1%)	198 (1.9%)	1 (Reference)	1 (Reference)	1 (Reference)
Middle	8,358 (97.3%)	241 (2.7%)	1.41 (1.05–1.88)	1.38 (1.02–1.87)	1.36 (1.01–1.85)
Lowest	8,360 (96.0%)	380 (4.0%)	2.09 (1.58–2.77)	1.82 (1.34–2.47)	1.88 (1.39–2.55)

Results may not add due to missing values. Percentages are shown in rows.

^a^ Model adjusted for gender, age, education level, socioeconomic status, marital status, region, and place of residence

^b^ Model adjusted for gender, age, education level, socioeconomic status, marital status, region, place of residence, daily smoking, binge drinking, previous depression, and hypertension status.

## Discussion

### Main findings

According to our results, there is evidence of a strong association between the consumption of fruits and/or vegetables and depressive symptoms after controlling for potential confounders. Moreover, lower consumption of fruits and/or vegetables was associated with greater prevalence of depressive symptoms, although this trend was not completely clear in the case of vegetables. Interestingly, only 3% of individuals in the study population reported symptoms consistent with clinical depression, which requires any kind of therapy; whereas <5% of the study population reported consuming ≥5 servings of fruits and vegetables per day according to the WHO recommendations.

### Comparison with other studies

Previous studies have reported similar findings that our study [9, 10, 26, 27]; however, certain considerations must be evaluated. For example, a study in Australian adults (18–74 years) found that increased consumption of certain foods, including meat, poultry and vegetables was associated with a lower probability of developing depressive symptoms [28]. However, participants reported self-diagnosis of depression rather than being directly evaluated by a screening tool. On the other hand, a cross-sectional study in China reported that consumption of vegetables but no of fruits was associated with lower depressive symptoms in individuals aged ≥65 years [29]. Moreover, the Geriatric Depression Scale was used in this survey instead of the PHQ-9 as in our study. Similarly, a study on Chinese college students showed that not only low consumption of fruit was associated with depressive symptoms, but other components of the diet (junk food, among others) also were associated with greater levels of depression [30].

Conversely, some studies have found that depressive symptoms were associated with poor quality diet [31, 32]. Given the cross-sectional nature of these studies, reverse causality may arise as a problem. However, data from prospective studies are also important. For example, a prospective cohort study, conducted in Spain, reported an inverse relationship between adherence to the Mediterranean diet and depression [26]. Moreover, there was a dose-response relationship between consumption of fruits, nuts and legumes, and the presence of depression. Apparently, these results suggest that some biochemical components of the Mediterranean diet might be associated with the reduction of depressive symptoms. Similarly, in a prospective cohort of individuals aged ≥65 years performed in Taiwan, including four years of follow-up, found that both, fruits and vegetables, reduced the risk of depression [27]. Thus, our results are consistent with those reported by longitudinal studies.

Although the trend effect of vegetables intake was not clear in our study, other reports have found that some components, mainly sulforaphane, can have an effect on depressive symptoms. Thus, a relatively recent study reported that the sulforaphane, present in cruciferous vegetables such as broccoli sprout, may prevent the onset of depression related to chronic inflammation [33]. Similarly, the dietary intake of glucoraphanin, a precursor of sulforaphane, appears to increase resilience to stress in mice [34]. Unfortunately, data regarding the kind of fruits or vegetables consumed by participants was not available. Thus, more studies are needed to clarify what components of fruits and vegetables are beneficial to mental health.

### Other relevant results

Less than 5% of participants consumed the WHO recommended consumption of fruits and vegetables (≥5 servings per day). This finding is relevant because a greater consumption of fruits and vegetables is associated with several potential benefits in health, including better mental health [11], but also reduction in the risk of mortality for other non communicable diseases such as cardiovascular disease and cancer [35]. In a previous study evaluating the intake of fruits and vegetables in 52 low- and middle-income countries reported the around 80% of individuals consumed less than the minimum consumption recommended by the WHO [36].

Regarding depression, only 3% presented symptoms consistent with the need of treatment according to the PHQ-9. A previous study enrolling individuals from 18 to 65 years from 5 of the most important cities from Peru reported a lifetime prevalence of depression of 6.4% [4]. These differences might be explained by the use of different tools to assess depression but also because older people as well as rural areas were not included in the aforementioned study. In addition, a recent systematic review found a global point prevalence of major depressive disorder of 4.7% after controlling for methodological differences [37]. This review also found 13 studies on the prevalence of depression in South America, reporting a prevalence of 4% in the region, very close to our estimates.

### Public health relevance

The low consumption of fruits and vegetables in our community can help implement appropriate strategies to encourage their consumption. Peru is a producer of a variety of fruits and vegetables which can be easily accessible, and therefore there is a need of implementing appropriate strategies to ensure a diet of quality at the population level. An appropriate intake of fruits and vegetables may help reduce mental health problems as well as cardiovascular disease [38–40]. Thus, consumption of fruits and vegetables at the population level should start from childhood. As these food groups are essential for proper child development, their consumption should be encouraged from an early age, guarantying their intake in the long term [41]. Given the nutritional and epidemiological transition that several low- and middle-income countries [42], including Peru, are undergoing, this kind of initiative is critical.

### Strengths and limitations

Strengths of this study include a multi-stage sampling, representative and with an adequate sample size to ensure an appropriate response to the proposed research aims. However, this study has some limitations. First, given the cross-sectional nature of the study, it can evaluate only association and not causality. Moreover, there is risk of reverse causality in our findings. However, our results are in line with existing longitudinal research [26, 27]. Second, information on the consumption of fruits and vegetables was self-reported based on the previous seven days before the application of the questionnaire. Moreover, information about the type of fruits and vegetables consumed by participants was not available. In addition, although small, some recall and social desirability bias might arise. However, the WHO STEPs, a well-known tool for measuring the consumption of fruits and vegetables, was used. In addition, any overestimation of the fruits and vegetables consumption would reduce the probability of reject the null hypothesis of no association. Third, our results might be biased due to the absence of other potential confounders such as physical activity, family history of depression, stress, co-morbidities, etc. In addition, other diet patterns, including the consumption of omega-3 fatty acids, known to prevent depressive symptoms [43], was not available in the database. Finally, the PHQ-9 is a screening and not a diagnostic tool for depression. However, a conservative cutoff was used to include those with depressive symptoms requiring medication or psychological management, which, in theory, would reduce the risk of misclassification.

## Conclusions

This study found an inverse relationship between consumption of fruits and/or vegetables and the presence of depressive symptoms. Moreover, less than 5% of subjects reported consuming fruits and vegetables as recommended by the WHO. Around 3% of individuals had depressive symptoms assessed by the PHQ-9. There is a need to implement appropriate strategies to promote better diet patterns with potential impact on mental health.

## Supporting information

S1 Table Characteristics of the study population according to fruits and vegetables consumption. (DOCX)

S2 Table Characteristics of the study population according to depressive symptoms. (DOCX)

S3 Table Association between fruits and vegetables consumption and depressive symptoms: Crude and adjusted models. (DOCX)
