# Supplementary material for: Fruits and vegetables consumption and depressive symptoms: A population-based study in Peru
Source: PLoS One. 2017 Oct 12;12(10):e0186379. doi: 10.1371/journal.pone.0186379 (PMC5638500; doi:10.1371/journal.pone.0186379)
Supplement: S1 Table — (DOCX) [file pone.0186379.s001.docx]

# S1 Table. Characteristics of the study population according to fruits and vegetables consumption

|  | **Fruits and vegetables consumption (in tertiles)** | | |  |
| --- | --- | --- | --- | --- |
|  | **Highest** | **Middle** | **Lowest** | **p-value^a^** |
|  | **(n = 8,509)** | **(n=8599)** | **(n=8740)** |  |
| ***Gender*** |  |  |  | < 0.001 |
| Male | 3,517 (41.3%) | 4,012 (46.7%) | 4,375 (50.1%) |  |
| Female | 4,992 (58.7%) | 4,587 (53.3%) | 4365 (49.9%) |  |
| ***Age*** |  |  |  | < 0.001 |
| 18 – 34 years | 3,107 (38.2%) | 3,018 (36.8%) | 2,609 (31.2%) |  |
| 35 – 54 years | 3,074 (37.8%) | 3,083 (37.5%) | 2,850 (34.1%) |  |
| 55 – 74 years | 1,559 (19.1%) | 1,642 (20.0%) | 2,037 (24.3%) |  |
| 75+ years | 401 (4.9%) | 470 (5.7%) | 874 (10.4%) |  |
| Missing values | 368 | 386 | 370 |  |
| ***Education level*** |  |  |  | < 0.001 |
| < 7 years | 2,383 (28.1%) | 3,017 (35.2%) | 4,483 (51.4%) |  |
| 7- 11 years | 3,323 (39.1%) | 3,259 (38.0%) | 2,776 (31.9%) |  |
| 12+ years | 2,787 (32.8%) | 2,299 (26.8%) | 1,452 (16.7%) |  |
| Missing values | 16 | 24 | 29 |  |
| ***Socioeconomic status*** |  |  |  | < 0.001 |
| Low | 2,034 (23.9%) | 2,516 (29.3%) | 4,213 (48.2%) |  |
| Middle | 2,843 (33.4%) | 3,112 (36.2%) | 2,707 (31.0%) |  |
| High | 3,632 (42.7%) | 2,971 (34.6%) | 1,820 (20.8%) |  |
| ***Marital status*** |  |  |  | <0.001 |
| Married | 5,372 (63.1%) | 5,472 (63.6%) | 5,389 (61.7%) |  |
| Never married | 1,674 (19.7%) | 1,641 (19.1%) | 1,485 (17.0%) |  |
| Previously married | 1,463 (17.2%) | 1,486 (17.3%) | 1,866 (21.3%) |  |
| ***Region*** |  |  |  | < 0.001 |
| Coastal | 3,855 (45.3%) | 3,483 (40.5%) | 2,712 (31.0%) |  |
| Highlands | 2,712 (31.9%) | 3,498 (40.7%) | 4,430 (50.7%) |  |
| Jungle | 1,942 (22.8%) | 1,618 (18.8%) | 1,598 (18.3%) |  |
| ***Place of residence*** |  |  |  | < 0.001 |
| Urban | 6,010 (70.6%) | 5,569 (64.8%) | 4,343 (49.7%) |  |
| Rural | 2,499 (29.4%) | 3,030 (35.2%) | 4,397 (50.3%) |  |
| ***Daily smoking*** |  |  |  | 0.22 |
| No | 8,135 (97.7%) | 8,430 (98.1%) | 8,540 (97.8%) |  |
| Yes | 192 (2.3%) | 165 (1.9%) | 196 (2.2%) |  |
| Missing values | 2 | 4 | 4 |  |
| ***Binge drinking*** |  |  |  | 0.04 |
| No | 7,477 (88.0%) | 7,581 (88.3%) | 7,776 (89.2%) |  |
| Yes | 1,022 (12.0%) | 1,008 (11.7%) | 945 (10.8%) |  |
| Missing values | 10 | 10 | 19 |  |
| ***Previous depression*** |  |  |  | <0.001 |
| No | 8,120 (95.5%) | 8,290 (96.4%) | 8,500 (97.3%) |  |
| Yes | 386 (4.5%) | 306 (3.6%) | 236 (2.7%) |  |
| Missing values | 3 | 3 | 4 |  |
| ***Hypertension status*** |  |  |  | 0.81 |
| No | 6,582 (77.6%) | 6,682 (78.0%) | 6,766 (77.6%) |  |
| Yes | 1,899 (22.4%) | 1,887 (22.0%) | 1,950 (22.4%) |  |
| Missing values | 28 | 30 | 24 |  |

^a^ P-value was calculated using Chi squared test
