# Supplementary material for: Fruits and vegetables consumption and depressive symptoms: A population-based study in Peru
Source: PLoS One. 2017 Oct 12;12(10):e0186379. doi: 10.1371/journal.pone.0186379 (PMC5638500; doi:10.1371/journal.pone.0186379)
Supplement: S2 Table — (DOCX) [file pone.0186379.s002.docx]

# S2 Table: Characteristics of the study population according to depressive symptoms

|  | **Depressive symptoms** | |  |
| --- | --- | --- | --- |
|  | **No** | **Yes** | **p-value^a^** |
|  | **(n = 25,029)** | **(n=819)** |  |
| ***Gender*** |  |  | < 0.001 |
| Male | 11,683 (98.1%) | 221 (1.9%) |  |
| Female | 13,346 (95.7%) | 598 (4.3%) |  |
| ***Age*** |  |  | < 0.001 |
| 18 – 34 years | 8,584 (98.3%) | 150 (1.7%) |  |
| 35 – 54 years | 8,776 (97.4%) | 231 (2.6%) |  |
| 55 – 74 years | 4,992 (95.3%) | 246 (4.7%) |  |
| 75+ years | 1,594 (91.4%) | 151 (8.7%) |  |
| Missing values | 1,083 | 41 |  |
| ***Education level*** |  |  | < 0.001 |
| < 7 years | 9,379 (94.9%) | 504 (5.1%) |  |
| 7- 11 years | 9.142 (97.7%) | 216 (2.3%) |  |
| 12+ years | 6,447 (98.6%) | 91 (1.4%) |  |
| Missing values | 61 | 8 |  |
| ***Socioeconomic status*** |  |  | < 0.001 |
| Low | 8,390 (95.7%) | 373 (4.3%) |  |
| Middle | 8,401 (97.0%) | 261 (3.0%) |  |
| High | 8,238 (97.8%) | 185 (2.2%) |  |
| ***Marital status*** |  |  | < 0.001 |
| Married | 15,818 (97.4%) | 415 (2.6%) |  |
| Never married | 4,703 (98.0%) | 97 (2.0%) |  |
| Previously married | 4,508 (93.6%) | 307 (6.4%) |  |
| ***Region*** |  |  | < 0.001 |
| Coastal | 9,795 (97.5%) | 255 (2.5%) |  |
| Highlands | 10,200 (95.9%) | 440 (4.1%) |  |
| Jungle | 5,034 (97.6%) | 124 (2.4%) |  |
| ***Place of residence*** |  |  | <0.001 |
| Urban | 15,489 (97.3%) | 433 (2.7%) |  |
| Rural | 9,540(96.1%) | 386 (3.9%) |  |
| ***Daily smoking*** |  |  | 0.71 |
| No | 24,483 (96.8%) | 802 (3.2%) |  |
| Yes | 537 (97.1%) | 16 (2.9%) |  |
| Missing values | 9 | 1 |  |
| ***Binge drinking*** |  |  | <0.001 |
| No | 22,072 (96.7%) | 762 (3.3%) |  |
| Yes | 2,919 (98.1%) | 56 (1.9%) |  |
| Missing values | 38 | 1 |  |
| ***Previous depression*** |  |  | <0.001 |
| No | 23,043 (97.1%) | 674 (2.9%) |  |
| Yes | 787 (89.2%) | 94 (10.8%) |  |
| Missing values | 10 | 0 |  |
| ***Hypertension status*** |  |  | <0.001 |
| No | 19,494 (97.3%) | 536 (2.7%) |  |
| Yes | 5,458 (95.2%) | 278 (4.8%) |  |
| Missing values | 77 | 5 |  |

^a^ P-value was calculated using Chi squared test
