# Supplementary material for: Fruits and vegetables consumption and depressive symptoms: A population-based study in Peru
Source: PLoS One. 2017 Oct 12;12(10):e0186379. doi: 10.1371/journal.pone.0186379 (PMC5638500; doi:10.1371/journal.pone.0186379)
Supplement: S3 Table — (DOCX) [file pone.0186379.s003.docx]

# S3 Table: Association between fruits and vegetables consumption and depressive symptoms: Crude and adjusted models

|  | **Depressive symptoms** | | **Crude model** | **Adjusted model^a^** | **Adjusted model^b^** |
| --- | --- | --- | --- | --- | --- |
| ***Consumption of...*** | **No (n = 23,839)** | **Yes (n=768)** | **PR (95%CI)** | **PR (95%CI)** | **PR (95%CI)** |
| ***...fruits*** |  |  |  |  |  |
| Highest | 7,724 (97.6%) | 187 (2.4%) | 1 (Reference) | 1 (Reference) | 1 (Reference) |
| Middle | 8,600 (97.1%) | 258 (2.9%) | 1.23 (1.02 – 1.48) | 1.09 (0.90 – 1.33) | 1.14 (0.94 – 1.38) |
| Lowest | 8,705 (95.9%) | 374 (4.1%) | 1.74 (1.47 – 2.07) | 1.30 (1.08 – 1.57) | 1.37 (1.14 – 1.65) |
| ***...vegetables*** |  |  |  |  |  |
| Highest | 6,842 (97.5%) | 178 (2.5%) | 1 (Reference) | 1 (Reference) | 1 (Reference) |
| Middle | 9,729 (97.5%) | 244 (2.5%) | 0.96 (0.80 – 1.17) | 0.89 (0.73 – 1.08) | 0.90 (0.74 – 1.09) |
| Lowest | 8,458 (95.5%) | 397 (4.5%) | 1.77 (1.49 – 2.10) | 1.35 (1.12 – 1.64) | 1.41 (1.17 – 1.71) |
| ***...fruits and vegetables*** |  |  |  |  |  |
| Highest | 8,311 (97.7%) | 198 (2.3%) | 1 (Reference) | 1 (Reference) | 1 (Reference) |
| Middle | 8,358 (97.2%) | 241 (2.8%) | 1.20 (1.01 – 1.45) | 1.11 (0.91 – 1.34) | 1.13 (0.93 – 1.37) |
| Lowest | 8,360 (95.7%) | 380 (4.3%) | 1.87 (1.58 – 2.21) | 1.39 (1.15 – 1.68) | 1.45 (1.21 – 1.75) |

Results may not add due to missing values. Percentages are shown in rows.

^a^ Model adjusted for gender, age, education level, socioeconomic status, marital status, region, and place of residence

^b^ Model adjusted for gender, age, education level, socioeconomic status, marital status, region, place of residence, daily smoking, binge drinking, previous depression, and hypertension status.
